# Kineret^®^/IL-1ra Blocks the IL-1/IL-8 Inflammatory Cascade during Recombinant Panton Valentine Leukocidin-Triggered Pneumonia but Not during *S. aureus* Infection

**DOI:** 10.1371/journal.pone.0097546

**Published:** 2014-06-06

**Authors:** Delphine Labrousse, Magali Perret, Davy Hayez, Sonia Da Silva, Cédric Badiou, Florence Couzon, Michèle Bes, Pascal Chavanet, Gérard Lina, François Vandenesch, Delphine Croisier-Bertin, Thomas Henry

**Affiliations:** 1 Vivexia, Dijon, France; 2 Université de Lyon, Lyon, France; 3 INSERM U1111, Lyon, France; 4 CNRS, UMR 5308, Lyon, France; 5 Ecole Normale Supérieure, Lyon, France; 6 Infectious Diseases Department, University Hospital, Dijon, France; University Medical Center Utrecht, Netherlands

## Abstract

**Objectives:**

Community-acquired *Staphylococcus aureus* necrotizing pneumonia is a life-threatening disease. Panton Valentine Leukocidin (PVL) has been associated with necrotizing pneumonia. PVL triggers inflammasome activation in human macrophages leading to IL-1β release. IL-1β activates lung epithelial cells to release IL-8. This study aimed to assess the relevance of this inflammatory cascade in vivo and to test the potential of an IL-1 receptor antagonist (IL-1Ra/Kineret) to decrease inflammation-mediated lung injury.

**Methods:**

We used the sequential instillation of Heat-killed *S. aureus* and PVL or *S. aureus* infection to trigger necrotizing pneumonia in rabbits. In these models, we investigated inflammation in the presence or absence of IL-1Ra/Kineret.

**Results:**

We demonstrated that the presence of PVL was associated with IL-1β and IL-8 release in the lung. During PVL-mediated sterile pneumonia, Kineret/IL-1Ra reduced IL-8 production indicating the relevance of the PVL/IL-1/IL-8 cascade in vivo and the potential of Kineret/IL-1Ra to reduce lung inflammation. However, Kineret/IL-1Ra was ineffective in blocking IL-8 production during infection with *S. aureus*. Furthermore, treatment with Kineret increased the bacterial burden in the lung.

**Conclusions:**

Our data demonstrate PVL-dependent inflammasome activation during *S.aureus* pneumonia, indicate that IL-1 signaling controls bacterial burden in the lung and suggest that therapy aimed at targeting this pathway might be deleterious during pneumonia.

## Introduction

The prevalence of community-acquired *Staphylococcus aureus* pneumonia is low, but the disease can be very severe, with lethality higher than 40% in children and young adults [1], [2]. Due to the spread of community-acquired methicillin-resistant *S. aureus* (CA-MRSA) and the increased resistance of these strains to antibiotics, it is crucial to understand the pathophysiological mechanisms at play during severe CA-*S. aureus* pneumonia and to find novel therapeutic options.

Panton Valentine Leukocidin (PVL) is a bi-component leukotoxin composed of LukS-PV and LukF-PV. PVL is very cytotoxic to human neutrophils, monocytes and macrophages. Furthermore, PVL triggers the production of IL-8 [3] by neutrophils and of IL-1β by monocytes and macrophages [4], [5]. We have recently shown that IL-1β released by rPVL-intoxicated macrophages activates lung epithelial cells to release large amounts of IL-8. IL-1β and IL-8 are key cytokines to recruit neutrophils [6]. This inflammatory cascade could thus contribute to acute lung inflammation observed during infection. While inflammation is important to clear bacteria, it can be detrimental to the host by triggering tissue damage. Indeed, Diep et al. demonstrated that PVL was associated with increased inflammation and neutrophil recruitment, both of which trigger lung injury [7].

Kineret, also known as Anakinra, is a drug used to treat rheumatoid arthritis and several inflammasome-related diseases [8]. Kineret is a recombinant form of the naturally occurring IL-1 receptor antagonist (IL-1Ra). Kineret competes with the IL-1 receptor for the binding of IL-1α and IL-1β. The safety of Kineret is well-characterized, thus allowing the drug to be used to treat other diseases [9].

In this work, we first characterized IL-8 secretion by human neutrophils, macrophages and lung epithelial cells in response to PVL and toxin-containing bacterial supernatant in vitro. We then performed an in vivo study with two specific aims: i) To assess whether inflammasome activation and the rPVL/IL-1/IL-8 inflammatory cascade were relevant during pneumonia. ii) To test whether Kineret/IL-1Ra could block this cascade and alleviate lung inflammation and injury.

Our results confirmed that PVL is a virulence factor that contributes to lung inflammation. Furthermore, we demonstrated that the instillation of heat-killed *S. aureus* (HKS) and rPVL, Kineret/IL-1ra reduced PVL-mediated IL-8 secretion, thus indicating the functionality of the rPVL/IL-1/IL-8 cascade in vivo. However, during infection with PVL^+^ *S. aureus*, we found that Kineret/IL-1ra had no effect on IL-8 levels, suggesting that other inflammatory mechanisms were at play. Finally, treatment with Kineret/IL-1ra increased bacterial replication in the lung, indicating that the IL-1 inflammatory pathway contributed to bacterial clearance. This latter result highlights the possible caveat of targeting inflammatory pathways and indicates that if such a therapeutic option were chosen, it could only be applied in the presence of a potent adjunctive antibiotic therapy.

## Materials and Methods

### Ethics Statement

The experimental protocol was approved by the local ethics committee for animal experimentation (Burgundy, Dijon, France-protocol number CE 1311). Blood packs from healthy donors were obtained anonymously through an agreement (InsermU1111/11_02_2013) with the Etablissement Français du Sang (EFS, Lyon, France) in accordance with French laws #98-535 and 99-1143 and with the principles of the Declaration of Helsinki. Each donor provided written informed consent, which is available upon request. The human cell lines (THP-1 and A549) used in this study were previously described [4].

### Bacterial strains, culture conditions and reagents

The MRSA *Staphylococcus aureus* USA 300 PVL^+^ Los Angeles Clone 0114 (hereafter termed USA300 LAC), SF8300 (BD425) and their isogenic PVL^−^ derivative strains (HT20060753 and BD0452) kindly provided by Franck DeLeo [10] and Binh Diep [7] were grown in CCY broth, which promotes the production of leukocidins. The clinical MRSA strain ST20120376 (a clonal complex CC121 PVL^+^ strain) was isolated from a patient suffering from necrotizing pneumonia. Supernatant was prepared from a 16 h culture and diluted 625 times before addition to cells. rPVL was produced and purified as previously described [4].

### Cells, culture conditions and in vitro tests

Human neutrophils were purified as previously described [11]. When applicable, Heat-killed *S. aureus* (HKS MOI 100∶1) and Kineret were added to the neutrophils 3 h before rPVL intoxication.

THP-1 cells and A549 cells were cultured as previously described [4]. For the mixed culture experiment, 10^5^ A549 cells were added to 10^3^ PMA-differentiated THP-1 cells 24 h before the addition of PVL. HKS was added for 16 h before cell intoxication.

### Production and treatment of necrotizing pneumonia in rabbits

#### Animals

Male New Zealand rabbits were bred and housed at the Zootechnical Center (University of Burgundy, Dijon, France) in accordance with current European Institute of Health guidelines.

#### Inoculum

USA 300 LAC and its isogenic PVL mutant were grown in CCY medium supplemented with pyruvic acid for 10 hours. The inoculum was adjusted to 3×10^9^ CFU/mL.

#### Rabbit Model of Necrotizing Pneumonia

**Figure 1:**
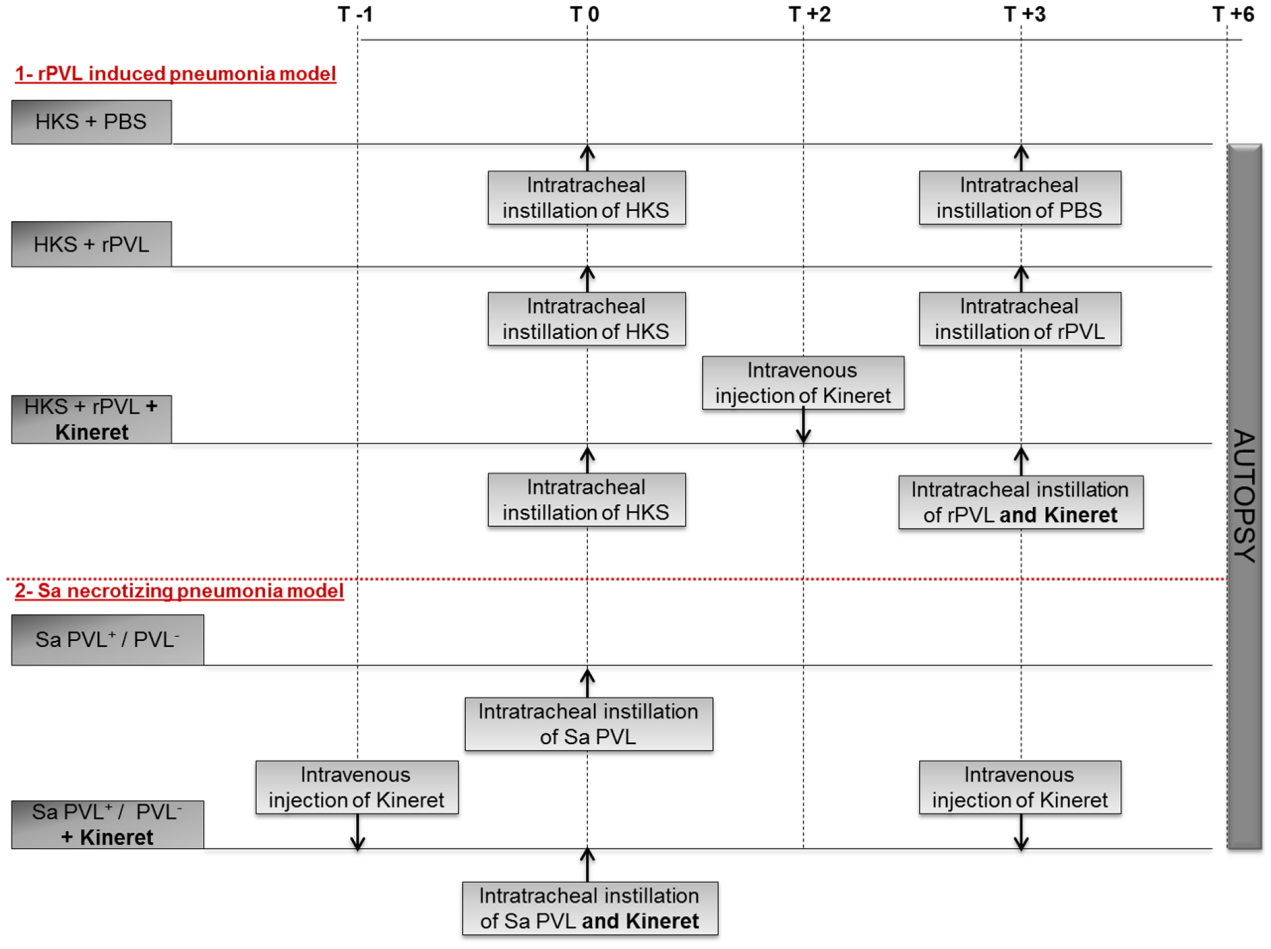
Study protocol.

The central venous catheters were installed and the pneumonia model was established as previously described [12], [13]. Bacterial pneumonia was induced by endobronchial challenge with 0.5 mL of saline containing 3×10^9^ CFU/mL. rPVL-induced pneumonia was achieved by endotracheal instillation of 12 µg each of LukS-PV and LukF-PV 3 hours after the delivery of 0.5 mL of HKS. Kineret/IL-1Ra was kindly provided EXW by SOBI^©^ and administered at doses of 10 mg/kg (Fig. 1).

### Evaluation of necrotizing pneumonia

#### Bacterial counts

The rabbits were euthanized 6 hours after challenge (Fig. 1). Each pulmonary lobe was weighed and homogenized in 5 mL of sterile saline for bacterial counts. Bacterial concentrations in each lobe were determined after adjusting for weight. The threshold value was 1 log_10_ CFU/g.

#### Macroscopic scoring

Macroscopic pulmonary injury scores were calculated according to a macroscopic scoring grid as previously described [14].

#### Bronchoalveolar Lavage Fluid

At the time of euthanasia (6 h after challenge), the lungs were excised and bronchoalveolar lavage (BAL) was performed on both inferior lobes by the instillation of 4 ml physiological saline. Total protein concentration in the BAL fluid (BALF) was determined by the Bradford protein assay according to the manufacturer's instructions (Pierce).

#### Measurements of IL-1β and IL-8

Concentrations of rabbit IL-1β and IL-8 in the BALF and in the crude homogenate of each pulmonary lobe were assessed by ELISA (Euromedex). The release of human IL-1β and IL-8 was quantified by ELISA using DuoSet ELISA kits (R&D systems, Lille, France).

#### Statistical analysis

In vitro and in vivo data were analyzed by an unpaired t-test and Mann-Whitney analysis, respectively, using Prism software (GraphPad, San Diego, USA). For in vitro data, means and standard deviations are shown. Two tailed p values are shown: n.s.: not significant, * p<0.05; ** p<0.01; *** p<0.0001.

## Results

### Kineret/IL-1Ra blocks the IL-1β/IL-8 inflammatory cascade observed in a co-culture of human macrophages and lung epithelial cells exposed to *S. aureus* supernatant, but has no effect on PVL-mediated neutrophil response

**Figure 2:**
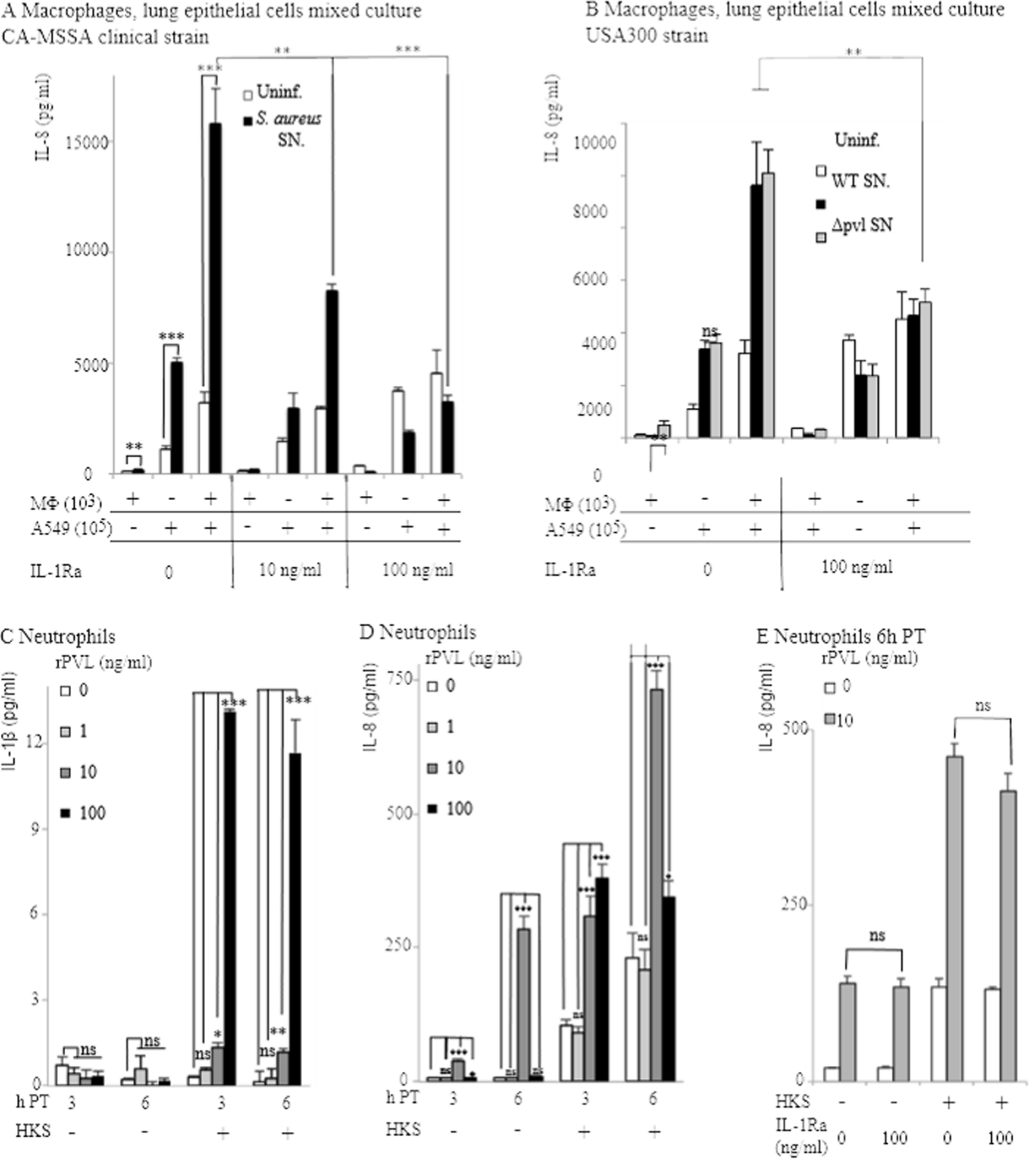
IL-8 produced in response to *S. aureus* supernatant by a co-culture of macrophages and lung epithelial cells is dependent on IL-1 signaling, while IL-8 produced by neutrophils is independent of IL-1. (A, B) THP-1 macrophages (10^3^ cells), A549 lung epithelial cells (10^5^) were cultured alone or mixed and treated as indicated with HKS, *S. aureus* CCY broth culture supernatant and Kineret/IL-1Ra. IL-8 was quantified by ELISA at 6 h post-intoxication. (C–E) As indicated, primary human neutrophils were treated with HKS, Kineret/IL-1Ra (E) and rPVL. IL-1β (C) and IL-8 levels were quantified at 6 h PI (E) or at the indicated time post-intoxication (C, D). One experiment representative of three independent experiments with three independent donors is shown.

Using a co-culture system of macrophages (1%) and lung epithelial cells (99%), we previously showed that very low concentrations of IL-1β released by rPVL-intoxicated macrophages triggered the release of large amounts of IL-8 by lung epithelial cells [4]. In line with this result, the addition of Kineret/IL-1Ra to a co-culture of macrophages (1%) and lung epithelial cells (99%) exposed to supernatant from a clinical PVL^+^ *S. aureus* strain isolated from a patient suffering from necrotizing pneumonia reduced IL-8 secretion in a dose-dependent manner (Fig. 2A). Though Kineret/IL-1Ra also reduced IL-8 levels in the same cellular model in response to supernatant from USA300 SF8300 [7] or LAC strains, we found no difference in IL-8 levels between WT USA300 strains and their isogenic ΔPVL mutants (Fig. 2B and data not shown). This result indicates that other staphylococcal secreted factors besides PVL can trigger the IL-1/IL-8 cascade. Altogether, these results suggested that the majority of IL-8 produced early on during infection with *S. aureus* could be secondary to IL-1 production triggered by the exposure of macrophages to PVL or other staphylococcal secreted factors. However, neutrophils are rapidly recruited to the lung and rapidly out-compete alveolar macrophages. We thus decided to quantify the production of IL-1β and IL-8 by human neutrophils treated with rPVL. In agreement with the low ability of human neutrophils to produce IL-1β [15], [16], [17], IL-1β production by rPVL-intoxicated primary human neutrophils was very low even when they were primed with HKS (Figure 2C [4]). As previously described [3], [18], human neutrophils intoxicated with rPVL at 10 µg/ml produced high levels of IL-8 (Fig. 2D). In contrast, rPVL at 100 µg/ml did not lead to consistent IL-8 production. This was probably due to the rapid death of neutrophils at this concentration [7], [11], [19], [20]. We then checked whether IL-8 production by neutrophils or by lung epithelial cells exposed to PVL-intoxicated neutrophils could be due to IL-1 signaling. Kineret/IL-1Ra had no impact on IL-8 production by neutrophils (Fig. 2E) or by a co-culture of neutrophils and lung epithelial cells (not shown), thus indicating that IL-8 production in neutrophils is independent of the IL-1/IL-8 cascade observed in macrophages ([4] and Fig. 2A). Altogether, these results indicated that PVL triggers different signaling pathways in human macrophages and neutrophils. Furthermore, these in vitro results suggested that Kineret/IL-1Ra could block IL-8 in vivo if the inflammatory response is driven by macrophages, with no major contribution from neutrophils.

### Infection with PVL^+^ *S. aureus* triggers IL-1β and IL-8 release in a rabbit model of necrotizing pneumonia

**Figure 3:**
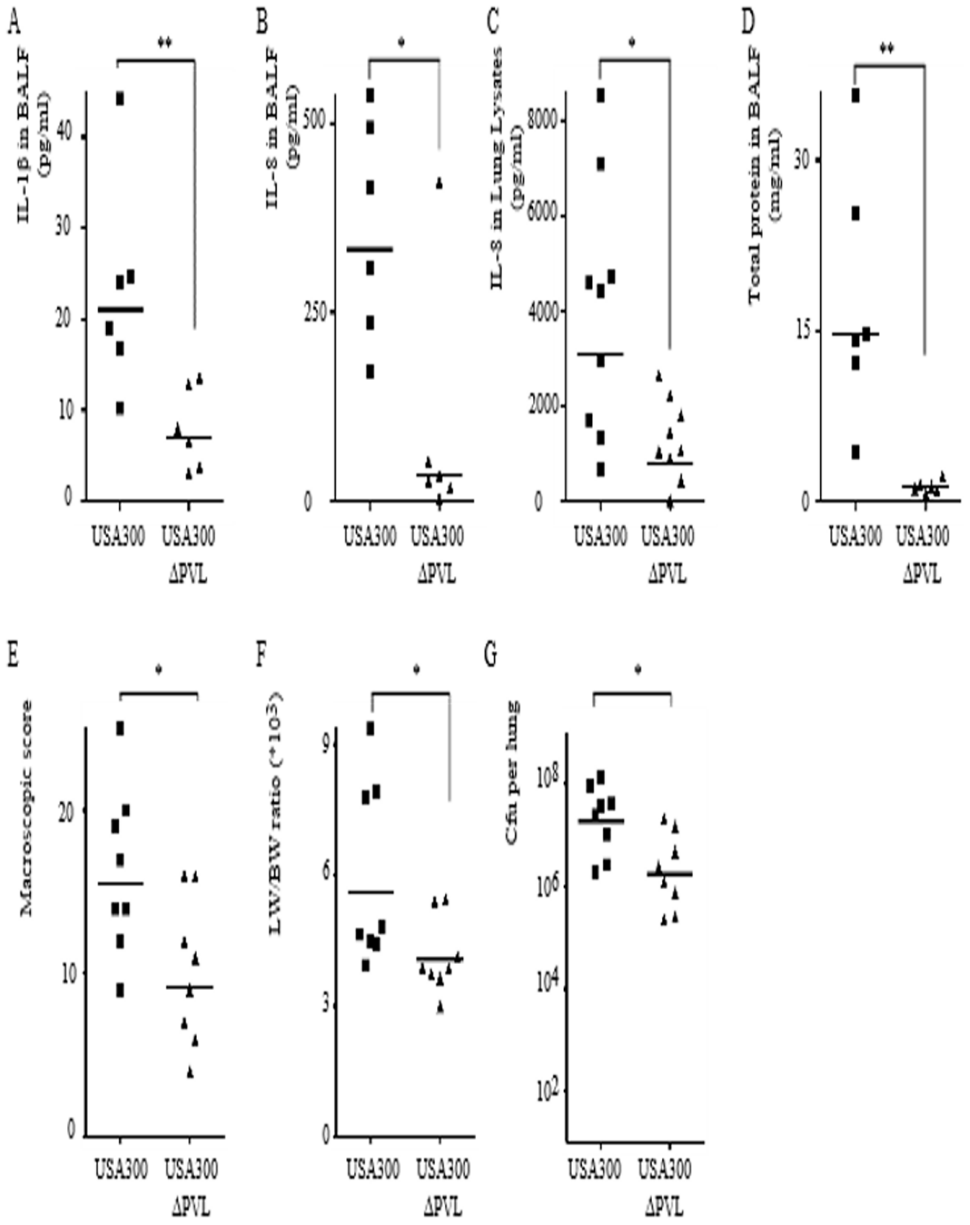
PVL is associated with an increase in IL-1β and IL-8 release in a rabbit model of *S. aureus*-mediated pneumonia. (A–G) Rabbits were inoculated intratracheally with 1.5×10^9^ cfu of USA300 LAC WT or ΔPVL strains and euthanized 6 h post-infection. IL-1β (A), IL-8 levels in BALF (B) or in lung lysates (C) were quantified by ELISA. (D) Total protein levels in BALF were quantified by a Bradford assay. (E) Gross pathological scoring was performed. (F) The ratio of lung weight to body weight is shown. (G) Bacterial burden as determined by CFU in each rabbit lung is shown. (A–D) Each point represents the value obtained in the BALF from one lobe or (E–G) the value obtained for one rabbit. (A–G) The geometric mean is shown. One experiment (n = 6) representative of two experiments (n = 12) is shown.

We and others have recently identified PVL as a major inflammasome activator in human monocytes and macrophages [4], [5]. However, whether PVL activates the inflammasome pathway in vivo remains to be determined. The rabbit model is a well established model to study PVL^+^ *S. aureus* diseases [7], [18], [20], [21], [22] and to discriminate between active and inactive anti-infective treatments [23] [13]. We thus investigated the role of PVL and its ability to activate the inflammasome pathway in a rabbit model of pneumonia [7]. Rabbits were inoculated intratracheally with 1.5×10^9^ CFU of the MRSA USA300 LAC strain or its isogenic ΔPVL mutant strain (Fig. 1). This infectious dose led to the rapid PVL-dependent death of infected rabbits (Figure S1), with macroscopic lesions of the lung at 15 h PI indicating necrosis (Figure S2). Necrotizing pneumonia is a fulminant disease. We therefore focused on early events. Infected animals were euthanized at 6 h PI. Bronchoalveolar lavage fluid (BALF) and lung lysates were collected to quantify cytokines. Infection with the PVL^+^ strain was associated with greater IL-1β release in the BALF than was the case with a ΔPVL mutant strain (Fig. 3A). This cytokine is notoriously difficult to detect in biological fluids [24] suggesting that the low levels of IL-1β detected in the BALF of PVL^+^ *S. aureus* infected rabbits were probably biologically relevant. As previously described [7], PVL^+^ *S. aureus*-mediated pneumonia was associated with a greater increase in IL-8 levels in the BALF (Fig. 3B) and the lung lysates than that observed during infection with the ΔPVL strain (Fig. 3C). This increase in inflammatory cytokines was associated with an increase i) in the permeability of the alveolar-capillary barrier as measured by the protein content of the BALF (Fig. 3D), ii) in the overall lung histopathology as determined by macroscopic scoring (Fig. 3E), iii) in the lung weight/body weight ratio (a measurement of pulmonary edema-Fig. 3F) and iv) in the lung bacterial burden (Fig. 3G).

### Sequential instillation of Heat-killed *S. aureus* and rPVL triggers pneumonia-like symptoms associated with IL-1β and IL-8 production

**Figure 4:**
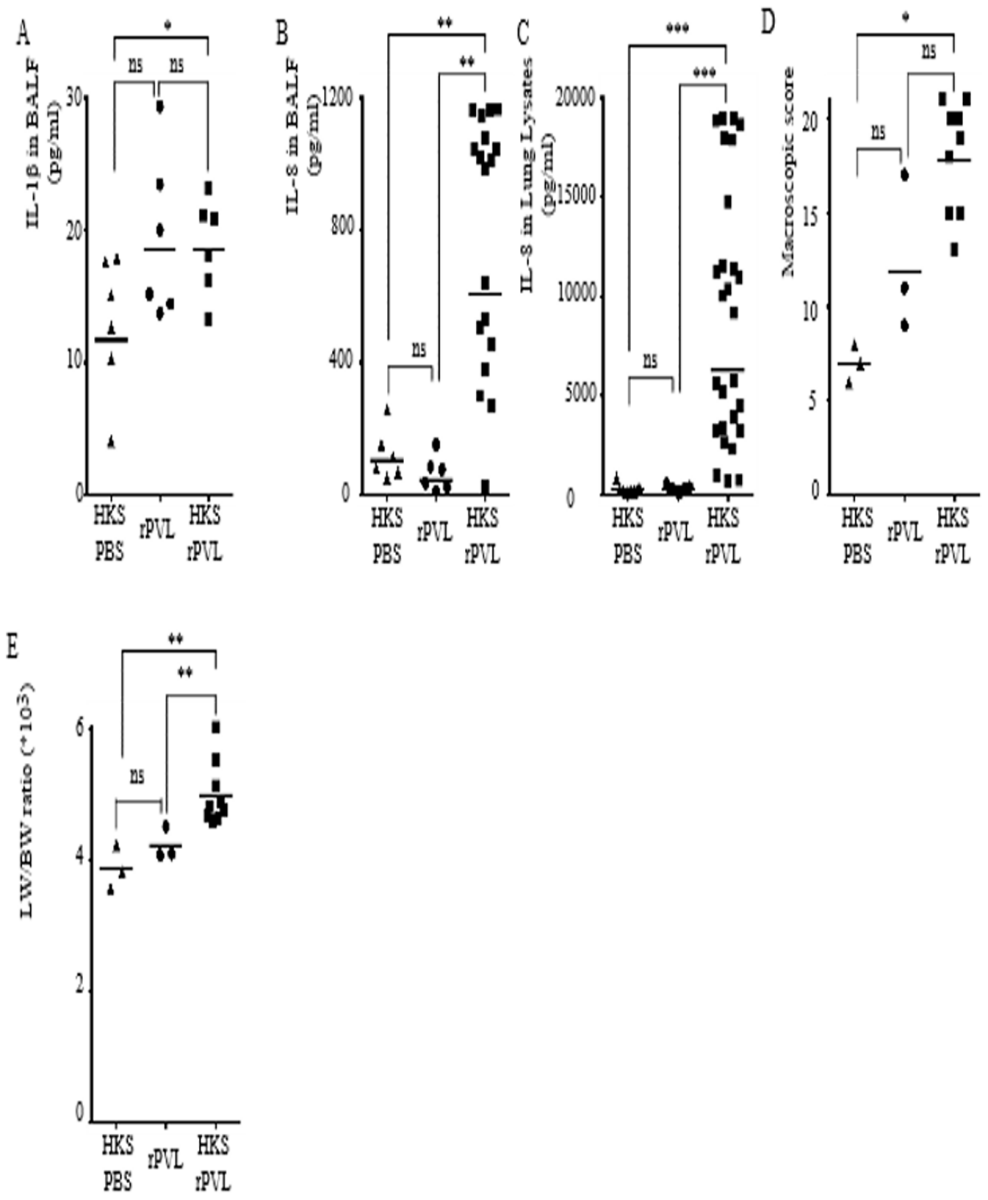
Sequential intratracheal instillation of HKS and rPVL triggers IL-1β and IL-8 release and reproduces some key aspects of *S. aureus*-mediated pneumonia. (A–E) Rabbits were inoculated intratracheally with HKS, 3 h later with PBS or with rPVL and euthanized 6 h post-HKS instillation. IL-1β (A) and IL-8 levels in BALF (B) or in lung lysates (C) were quantified by ELISA. (D) Gross pathological scoring was performed. (E) The ratio of lung weight on body weight is shown. (A–C) Each point represents the value obtained in the BALF from one lobe or (D, E) the value obtained for one rabbit. (A–F) The geometric mean is shown. Results from three independent experiments (n = 12) are shown.

To ensure that the differences in IL-1β and IL-8 production and lung injury was due to the presence of PVL, we investigated whether we could reproduce this inflammation using rPVL. In vitro, inflammasome activation in response to pore-forming toxins requires prestimulation with TLR agonists [25], [26]. We thus adapted the protocol established by Diep and collaborators [7] and instilled HKS followed 3 h later by rPVL intratracheally. Rabbits were euthanized 3 h post-rPVL instillation (Fig. 1). As described above for infection, in this sterile model, the presence of rPVL was associated with an increase in the levels of IL-1β (Fig. 4A) and IL-8 (Fig. 4B) in the BALF, and IL-8 (Fig. 4C) in lung lysates. Furthermore, the increase in inflammation mirrored an increase in the lung histopathology as determined by macroscopic scoring (Fig. 4D) and lung edema (Fig. 4E). Interestingly, at this time point, HKS alone had no effect on the secretion of cytokines, but worked in synergy with rPVL to induce IL-8 and pulmonary pathological features.

Altogether, these two models of HKS-rPVL-mediated and bacterial pneumonia indicated that the presence of PVL was associated in vivo with inflammasome activation and confirmed that PVL contributed to severe inflammation and lung injury in a rabbit model of necrotizing pneumonia [7], [18].

### Kineret/IL-1Ra blocks the PVL/IL-1/IL-8 inflammatory cascade observed in a HKS-rPVL-mediated pneumonia model

**Figure 5:**
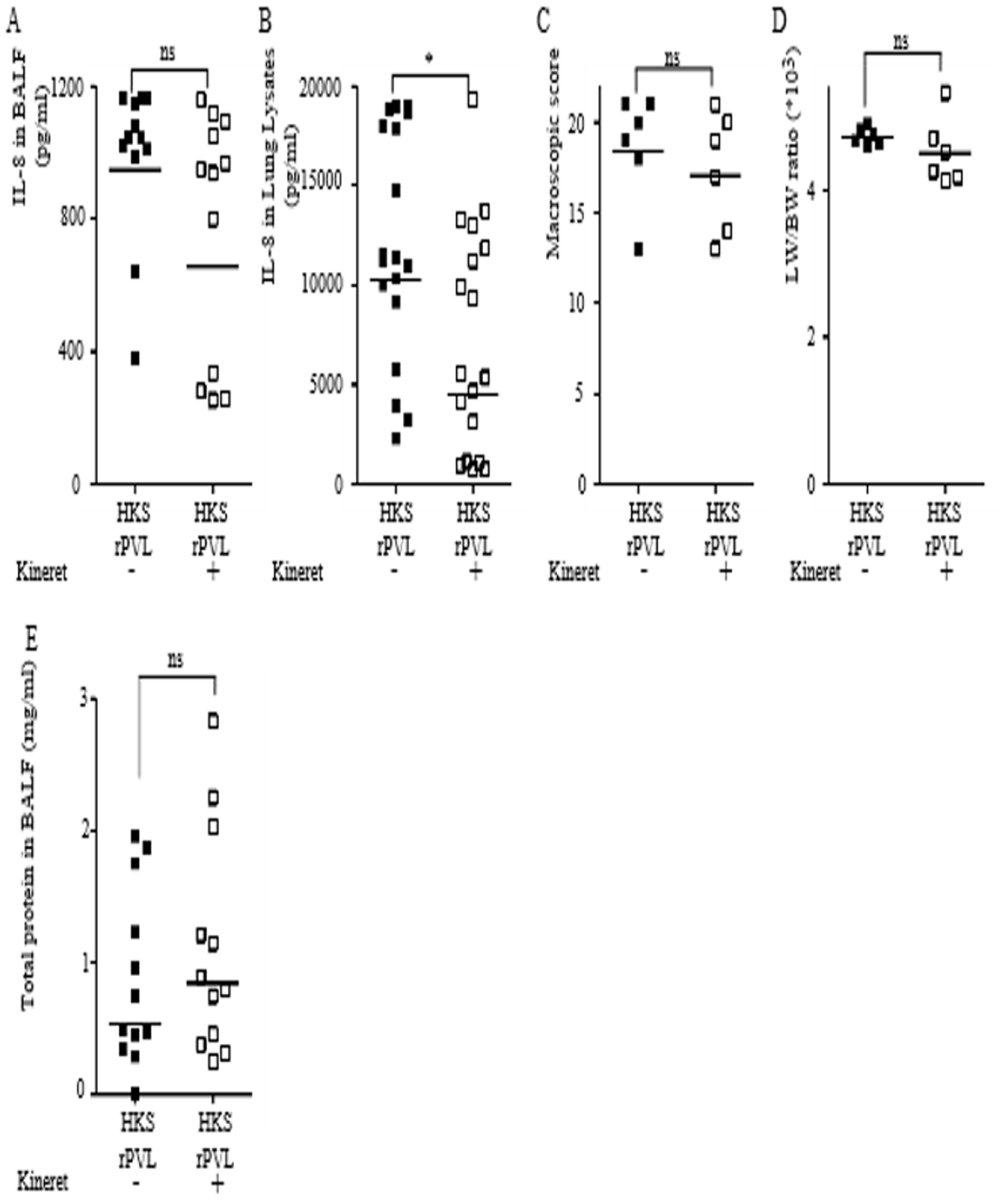
Kineret/IL-1Ra inhibits the IL-1/IL-8 cascade triggered by sequential intratracheal instillation of HKS and rPVL. (A–E) Rabbits were inoculated intratracheally with HKS, 3 h later with rPVL and euthanized 6 h post-HKS instillation. When applicable, Kineret/IL-1Ra was injected IV at 2 h post-HKS and was co-instillated with rPVL. IL-8 levels in BALF (A) or in lung lysates (B) were quantified by ELISA. (C) Gross pathological scoring was performed. (D) The ratio of lung weight to body weight is shown. (E) Total protein levels in BALF were quantified by Bradford assay. (A–B, E) Each point represents the value obtained in the BALF from one lobe or (C, D) the value obtained for one rabbit. (A–E) The geometric mean is shown. Results from two independent experiments (n = 12) are shown.

IL-1β and IL-8 are two key cytokines involved in the recruitment of neutrophils [6]. Neutrophils contribute to the pathology of necrotizing pneumonia [7]. We thus investigated whether Kineret/IL-1Ra was effective in vivo to decrease the IL-1/IL-8 inflammatory cascade and the inflammation-associated lung damages. We first tested Kineret/IL-1Ra on pneumonia triggered by sequential instillation of HKS and rPVL. To ensure the highest potency of this drug, we administered Kineret IV 2 h post-HKS treatment as well as in co-instillation with rPVL (Fig. 1). Kineret was used at 10 mg/kg [27]. Interestingly and as observed in vitro using human macrophages, treatment with Kineret reduced IL-8 levels in both BALF (Fig. 5A) and lung lysates (Fig. 5B). The reduction in IL-8 levels in lung lysate was much greater than in BALF possibly due to the poor bioavailability of IL-1Ra in the lung lumen. The inhibition of IL-1 signaling and IL-8 production did not decrease the macroscopic pathological score (Fig. 5C), edema formation (Fig. 5D) or the permeability of the alveolar-capillary barrier (Fig. 5E). Overall, these results demonstrated the presence of this PVL/IL-1/IL-8 cascade in vivo in the lung and showed that Kineret/IL-1Ra targets this pathway but has no detectable effect on lung pathophysiology.

### Kineret/IL-1Ra does not block the PVL/IL-1/IL-8 inflammatory cascade observed during lung infection with PVL^+^ *S. aureus*

**Figure 6:**
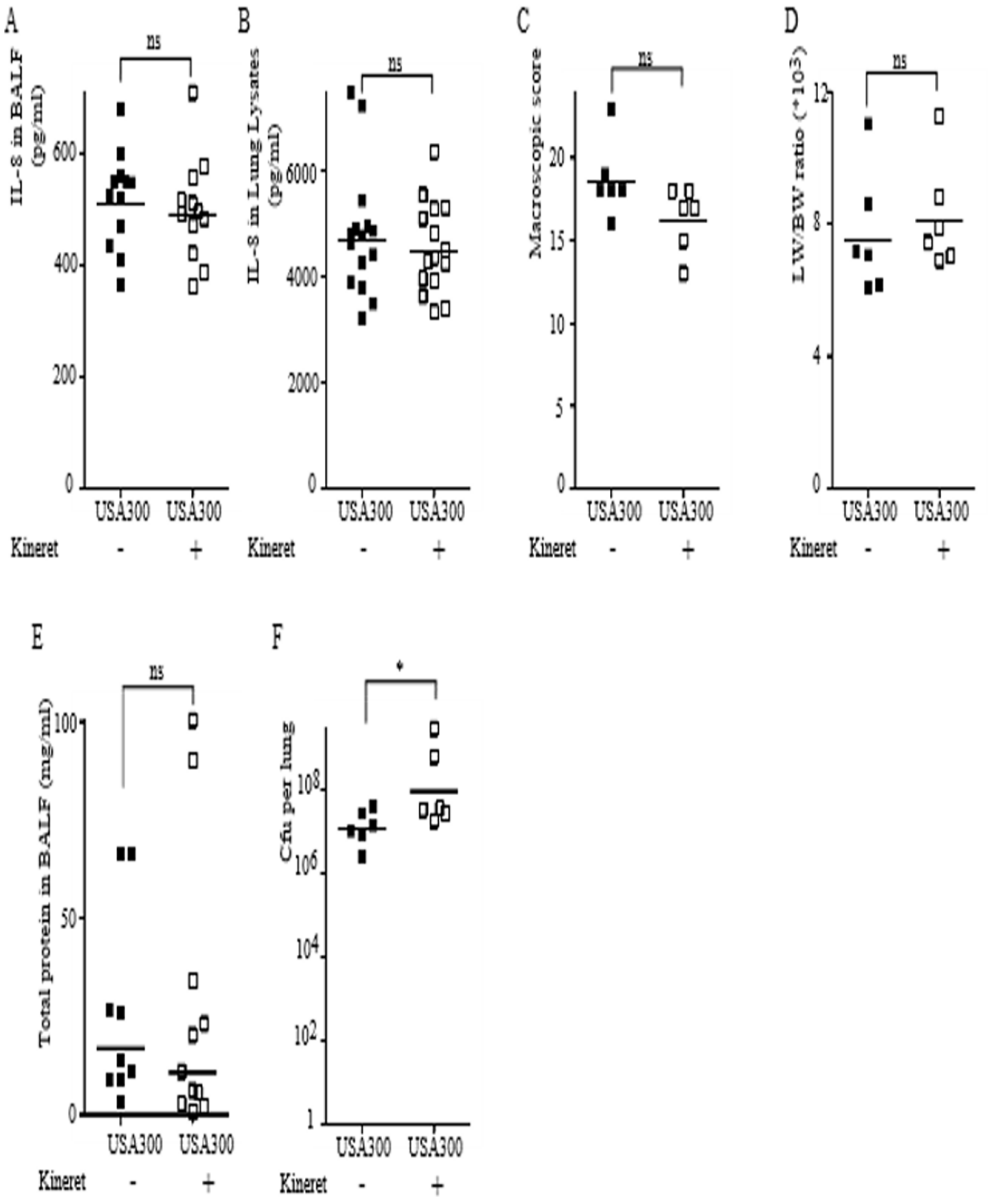
Kineret/IL-1Ra does not reduce IL-8 levels during PVL^+^ *S. aureus*-mediated pneumonia but increases lung bacterial load. (A–F) Rabbits were inoculated intratracheally with USA300 LAC strain and euthanized 6 h post-infection. When applicable, Kineret/IL-1Ra was injected IV 1 h before and 3 h after infection and was co-instilled with *S. aureus*. IL-8 levels in BALF (A) or in lung lysates (B) were quantified by ELISA. (C) Gross pathological scoring was performed. (D) The ratio of lung weight to body weight is shown. (E) Total protein levels in BALF were quantified by Bradford assay. (F) Bacterial burden as determined by CFU assay is shown. (A, B, E) Each point represents the value obtained in the BALF from one lobe or (C, D, F) the value obtained for one rabbit. (A–F) The geometric mean is shown. Results from two independent experiments (n = 12) are shown.

Since treatment with Kineret/IL-1Ra led to encouraging results on the possibility to target the IL-1/IL-8 cascade, we decided to investigate its potency during PVL^+^ *S. aureus*-mediated pneumonia (Fig. 1). In contrast to what we observed in the HKS-rPVL model, treatment with Kineret/IL-1Ra did not reduce IL-8 levels in either BALF (Fig. 6A) or lung lysates (Fig. 6B). As previously described in the HKS-rPVL model, treatment with Kineret/IL-1Ra was ineffective in reducing the pathological score (Fig. 6C), lung edema (Fig. 6D) or the permeability of the alveolar-capillary barrier (Fig. 6E). However, treatment with Kineret/IL-1Ra did result in a significant increase in the bacterial burden per lung (Fig. 6F) indicating that IL-1 signaling contributes to antibacterial defenses during necrotizing pneumonia.

## Discussion

CA-*S. aureus* necrotizing pneumonia is a severe disease with a high percentage of fatal outcomes. The role of PVL in experimental infections, in triggering specific human diseases or in affecting disease outcome is still debated [28], [29], [30]. Here, using infection or sequential instillations of HKS and rPVL, we confirmed the role of PVL in triggering inflammation and lung injury in a rabbit model of necrotizing pneumonia. Two recent studies have described the ability of PVL to activate the inflammasome in primary human monocytes and macrophages [4], [5]. We demonstrated in vivo that the presence of PVL was associated with an increase in IL-1β levels in the BALF of infected rabbits, thus highlighting the relevance of this pathway during pneumonia. Furthermore, we observed that the blockage of IL-1 signaling using Kineret/IL-1Ra led to a ten-fold increase in the bacterial burden in the lung of infected animals, indicating that IL-1 is critical for the antibacterial activity during acute pneumonia.

The current guidelines for the treatment of necrotizing pneumonia are the prompt and aggressive administration of toxin-suppressing antibiotics such as linezolid [23], [31], [32], [33]. Appropriate antibiotic therapy might not be sufficient in such fulminant diseases in which inflammation is intense and at least partly responsible for lung injury [7]. Indeed, numerous deaths have been observed in patients treated in a timely manner with effective antibiotic therapy [32], [34]. Adjunctive anti-inflammatory treatment is widely used in bacterial meningitis even though its efficacy might be limited [35]. The use of non-steroidal antiinflammatory drugs in CA pneumonia is associated with a more complicated course [36] and the use of dexamethasone in treating CA pneumonia is still debated [37], [38]. Furthermore, CA-pneumonia covers a wide range of diseases in terms of both the causative pathogenic agent and severity, thus highlighting the need to test adjunctive therapy in specific animal models of diseases. Here, we used a rabbit model of pneumonia to test the therapeutic potential of anti-inflammatory drugs. Due to the high sensitivity of rabbit neutrophils to PVL [20] and as demonstrated by others [7], [18], the rabbit is a good animal model to study PVL^+^-*S. aureus* pneumonia and to assess treatment efficacy. However, the limitations of our model include the high inoculum required to trigger robust pneumonia and the absence of an influenza-like viral infection preceding *S.aureus* infection, which is observed in most severe human clinical cases [1], [2]. We found that dexamethasone had no effect on IL-8 or any of the other parameters considered in our model of pneumonia (not shown). In contrast, we demonstrated that Kineret/IL-1Ra was effective in inhibiting the rPVL/IL-1/IL-8 cascade after sequential instillation of HKS and rPVL. Surprisingly, this effect was not observed during infection despite the detection of PVL-dependent IL-1β and IL-8 release. Several hypotheses can explain this difference: i) while the macrophage response may dominate in the sterile model (HKS+rPVL), a neutrophil-based response may be predominant during infection. Indeed, human neutrophils produced IL-8 in response to rPVL independently of IL-1 signaling (Fig. 2E). Furthermore, IL-8 levels were strongly reduced in neutropenic rabbits [7], [18]. ii) *S. aureus* and leukocidins/PSMs-intoxicated neutrophils release numerous proteases[39], [40] [41], which might degrade/inactivate Kineret/IL-1Ra. iii) Endogenous IL-1Ra, which is up-regulated during bacterial pneumonia [42], might mask the action of exogenously added IL-1Ra/Kineret.

In our sterile pneumonia model, despite the efficient block of IL-1 signaling and a reduction of 50% in IL-8 level, we found no reduction in neutrophil infiltration into the lung (not shown). This result suggests that neutrophil recruitment in the lung is based on several redundant pathways [6] and would require the targeting of numerous signaling pathways to be significantly reduced. Similarly, the failure of Kineret/IL-1ra clinical trials in severe sepsis led to the conclusion that strategies aimed at targeting a single inflammatory mediator were unlikely to work in such complex diseases. Importantly, such a lesson can be learned from *S. aureus*, which has evolved a large number of virulence factors targeting neutrophils [43], [44].

## Supporting Information

Figure S1 **Survival curves of LAC PVL and LAC ΔPVL-infected rabbits.**
(PPTX)

Figure S2 **Macroscopic Pulmonary Injury Scores of LAC PVL and LAC ÄPVL-infected rabbits.**
(PPTX)
