# Supplementary figures and images for: Kineret®/IL-1ra Blocks the IL-1/IL-8 Inflammatory Cascade during Recombinant Panton Valentine Leukocidin-Triggered Pneumonia but Not during S. aureus Infection
Source: PLoS One. 2014 Jun 6;9(6):e97546. doi: 10.1371/journal.pone.0097546 (PMC4048174; doi:10.1371/journal.pone.0097546)

## Slide 1
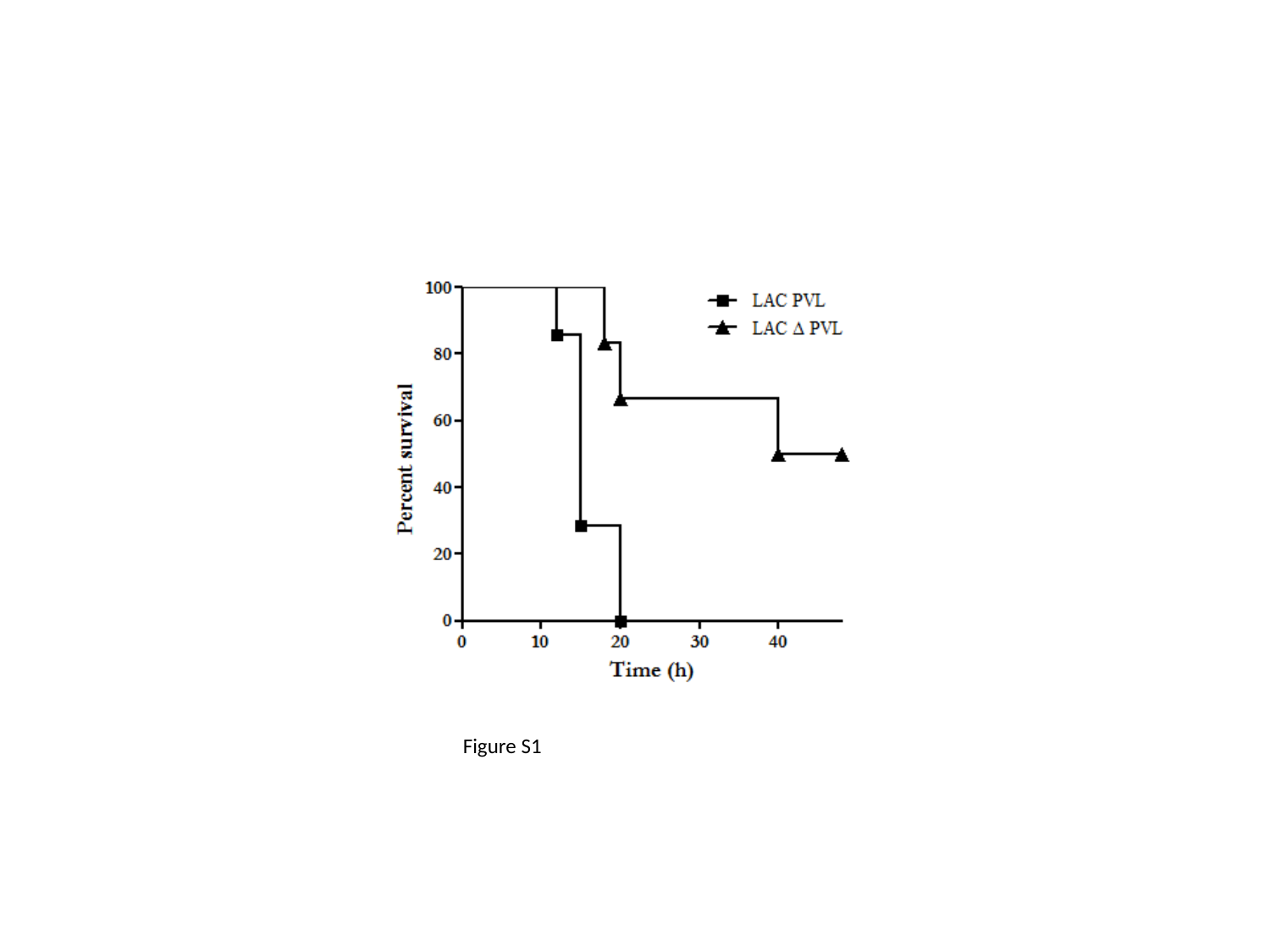

Figure S1

Supplement: Figure S1 — Survival curves of LAC PVL and LAC ΔPVL-infected rabbits. (PPTX) [file pone.0097546.s001.pptx]

## Slide 1
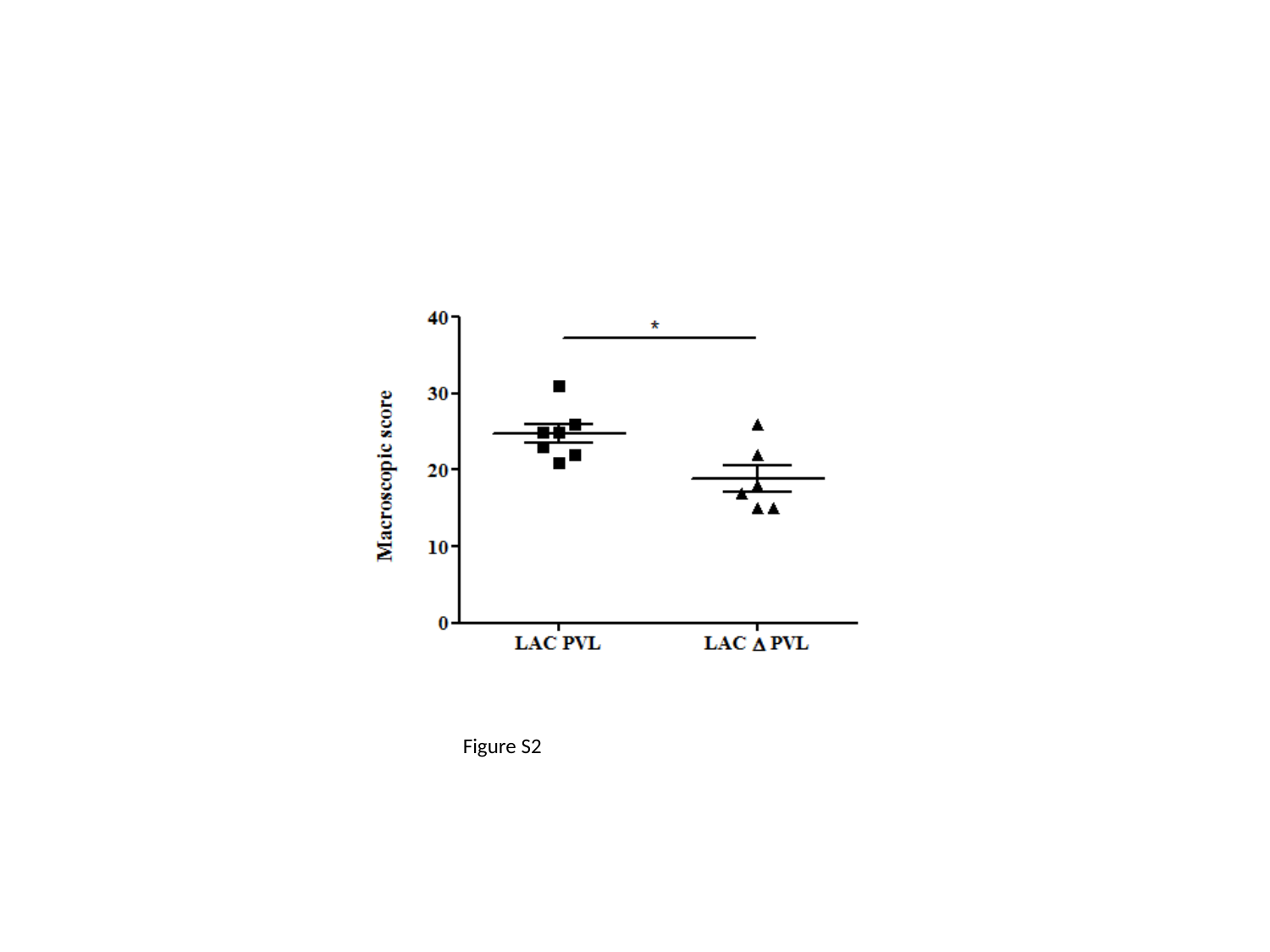

Figure S2

Supplement: Figure S2 — Macroscopic Pulmonary Injury Scores of LAC PVL and LAC ÄPVL-infected rabbits. (PPTX) [file pone.0097546.s002.pptx]
